# Anti-fibrotic efficacy of nintedanib in pulmonary fibrosis via the inhibition of fibrocyte activity

**DOI:** 10.1186/s12931-017-0654-2

**Published:** 2017-09-15

**Authors:** Seidai Sato, Shintaro Shinohara, Shinya Hayashi, Shun Morizumi, Shuichi Abe, Hiroyasu Okazaki, Yanjuan Chen, Hisatsugu Goto, Yoshinori Aono, Hirohisa Ogawa, Kazuya Koyama, Haruka Nishimura, Hiroshi Kawano, Yuko Toyoda, Hisanori Uehara, Yasuhiko Nishioka

**Affiliations:** 1 Department of Respiratory Medicine and Rheumatology, Graduate School of Biomedical Sciences, Tokushima University, 3-18-15 Kuramoto-cho, Tokushima, 770-8503 Japan; 2 National Hospital Organization Higashi Tokushima Medical Center, Tokushima, Japan; 3 Department of Pathology and Laboratory Medicine, Graduate School of Health Biosciences, Tokushima University, Tokushima, Japan

**Keywords:** Fibrocytes, Nintedanib, Pulmonary fibrosis

## Abstract

**Background:**

Nintedanib, a tyrosine kinase inhibitor that is specific for platelet-derived growth factor receptors (PDGFR), fibroblast growth factor receptors (FGFR), and vascular endothelial growth factor receptors (VEGFR), has recently been approved for idiopathic pulmonary fibrosis. Fibrocytes are bone marrow-derived progenitor cells that produce growth factors and contribute to fibrogenesis in the lungs. However, the effects of nintedanib on the functions of fibrocytes remain unclear.

**Methods:**

Human monocytes were isolated from the peripheral blood of healthy volunteers. The expression of growth factors and their receptors in fibrocytes was analyzed using ELISA and Western blotting. The effects of nintedanib on the ability of fibrocytes to stimulate lung fibroblasts were examined in terms of their proliferation. The direct effects of nintedanib on the differentiation and migration of fibrocytes were also assessed. We investigated whether nintedanib affected the accumulation of fibrocytes in mouse lungs treated with bleomycin.

**Results:**

Human fibrocytes produced PDGF, FGF2, and VEGF-A. Nintedanib and specific inhibitors for each growth factor receptor significantly inhibited the proliferation of lung fibroblasts stimulated by the supernatant of fibrocytes. Nintedanib inhibited the migration and differentiation of fibrocytes induced by growth factors in vitro. The number of fibrocytes in the bleomycin-induced lung fibrosis model was reduced by the administration of nintedanib, and this was associated with anti-fibrotic effects.

**Conclusions:**

These results support the role of fibrocytes as producers of and responders to growth factors, and suggest that the anti-fibrotic effects of nintedanib are at least partly mediated by suppression of fibrocyte function.

**Electronic supplementary material:**

The online version of this article (10.1186/s12931-017-0654-2) contains supplementary material, which is available to authorized users.

## Background

Platelet-derived growth factor (PDGF), fibroblast growth factor (FGF) 1/2, and vascular endothelial growth factor (VEGF) have been implicated in the pathogenesis of pulmonary fibrosis [1–5]. Nintedanib is a tyrosine kinase inhibitor that is specific also for PDGFRα and β, FGFR1, 2, and 3, and VEGFR1, 2, and 3 [6–8]. In two phase III clinical trials (INPULSIS 1 and 2), treatment with nintedanib for one year led to reductions in the annual rate of decline in forced vital capacity versus placebo in patients with idiopathic pulmonary fibrosis (IPF) [9]. However, the mechanisms by which nintedanib regulates pulmonary fibrosis is not fully explored. Several studies have reported the anti-fibrotic effects of BIBF 1000 [10], and nintedanib (BIBF1120) [7, 11]. However, the roles of main targets, PDGFR, FGFR and VEGFR of nintedanib have not yet been analyzed in detail.

Fibrocytes are monocyte-derived cells that are a subpopulation of mesenchymal progenitor cells [12]. Fibrocytes appear to be derived from the differentiation of CD14-positive peripheral blood mononuclear cells, and express markers of hematopoietic cells, leukocytes, and fibroblast products [12, 13]. Marked increases in circulating fibrocyte numbers and a positive correlation between the abundance of fibroblastic foci and the number of lung fibrocytes have been reported in patients with IPF [14, 15]. Moeller et al. also showed that the percentage of CD45/collagen-1-positive fibrocytes was increased in the peripheral blood of patients with IPF, and proposed that the quantification of circulating fibrocytes may allow for the prediction of early mortality in these patients [16]. These findings strongly suggest that fibrocytes are involved in the pathogenesis of pulmonary fibrosis. Furthermore, we previously indicated that the PDGF signaling pathway, which is a potential target for nintedanib, plays a critical role in fibrocyte migration into fibrotic lungs and contributes to fibrogenesis [17]. We also demonstrated that fibrocytes play a role in the pathogenesis of pulmonary fibrosis by producing various growth factors [18] (Abe S, et al. manuscript in preparation). However, the effects of nintedanib on fibrocytes remain unclear.

Therefore, we herein focus on fibrocytes and discuss several rationales for the anti-fibrotic properties of nintedanib. We assess the effects of nintedanib on the proliferation of fibroblasts induced by fibrocytes, the differentiation of fibrocytes from monocytes, and the migration of fibrocytes. We show that nintedanib reduces the number of fibrocytes that infiltrate in the lungs and mitigated fibrosis in an experimental murine model of pulmonary fibrosis.

## Methods

Detailed methods are described in the Additional file 1.

### Isolation of human fibrocytes and monocytes

Human fibrocytes were isolated according to previously described methods [17, 19]. All procedures for consent, sample collection, and privacy protection were approved by the Ethics Committee of Tokushima University Hospital. Human mononuclear cells (HMNC) were isolated from the peripheral blood of healthy volunteers, and cultured on fibronectin-coated dishes. After six to seven days, adherent cells were used as fibrocytes, the phenotype of which was confirmed by a flow cytometric analysis. Monocytes were isolated from HMNC with an automated magnetic cell separation device.

### Materials

Nintedanib and SB431542 were obtained from Boehringer Ingelheim GmbH & Co. KG (Biberach, Germany). SU5416, a VEGFR-specific inhibitor, was purchased from Abcam (Cambridge, MA). BGJ-398 and imatinib were purchased from Chemietek (Indianapolis, IN). Bleomycin (BLM) was purchased from Nippon Kayaku Co. (Tokyo, Japan).

### Measurement of growth factors

Mediator concentrations were measured in the cell culture supernatants of fibrocytes, monocytes, and fibroblasts using commercial enzyme-linked immunosorbent assay (ELISA) kits.

### Immunoblot analysis

Fibrocytes, monocytes, and fibroblasts were lysed and used for immunoblotting as described previously [20].

### Proliferation assay

MRC-5 cells were cultured in the cell culture supernatant of fibrocytes with various concentrations of inhibitors (0–1 μM) or recombinant growth factors (FGF2: 30 ng/ml, PDGF-AA: 100 ng/ml, PDGF-BB: 100 ng/ml, VEGF-A: 100 ng/ml) for 72 h. A [^3^H] thymidine deoxyribose (^3^H–TdR) incorporation assay was performed as described previously [3].

### Differentiation assay with recombinant growth factors

HMNC were seeded in fibronectin-coated 6-well plates with growth factor (FGF2: 30 ng/ml, PDGF-BB: 100 ng/ml, VEGF-A: 100 ng/ml), and various concentrations of inhibitors. Each growth factor and inhibitor was added again every 48 h. On day 6, attached cells were stained with Diff-Quick and counted.

### Cell migration assay

Fibrocytes were added to the upper chamber of cell culture inserts with a pore size of 8 μm in the presence or absence of various concentrations of nhibitors (0–100 nM). Growth factors (FGF2: 30 ng/ml, PDGF-BB: 100 ng/ml, VEGF-A: 100 ng/ml) were added to the lower chamber. After 20-h incubation, fibrocytes that had migrated to the bottom surface of the filter were stained with Diff-Quick and counted [17, 19].

### BLM-induced pulmonary fibrosis in mice

Eight-week-old C57BL/6 male mice were purchased from CLEA Japan (Tokyo, Japan). Mice received a single transbronchial instillation of 7.5 mg/kg BLM on day 0. Nintedanib at 60 mg/kg was administered daily by gavage until day 7. Lung tissue was analyzed on day 7 via a fluorescence-activated cell sorter (FACS) analysis and immunohistochemistry [17].

### Immunohistochemistry

Paraffin-embedded lung sections were stained with primary antibodies and then stained with fluorescence-conjugated secondary antibodies and 4′, 6-diamidino-2-phenylindole. Fluorescence images were captured with a confocal laser scanning microscope and counted [17].

### Facs

Minced lungs were digested, and the harvested cells were stained with antibodies for CD45, CXCR4 and collagen-1. Stained cells were analyzed using a FACScan flow cytometer (BD Biosciences-Pharmingen, San Diego, CA) [17].

### Statistical analysis

The significance of differences were analyzed using Mann–Whitney *U* test for unpaired samples, or a one-way ANOVA followed by a Dunnett’s test. Where appropriate, the Kruskal-Wallis H test was applied with Dunn’s test. *P* values of less than 0.05 were considered to be significant. Statistical analyses were performed using GraphPad Prism programme Ver. 5.01 (*GraphPad* Software Inc.).

## Results

### Comparison of growth factor expression among monocytes, fibrocytes, and fibroblasts

**Fig. 1:**
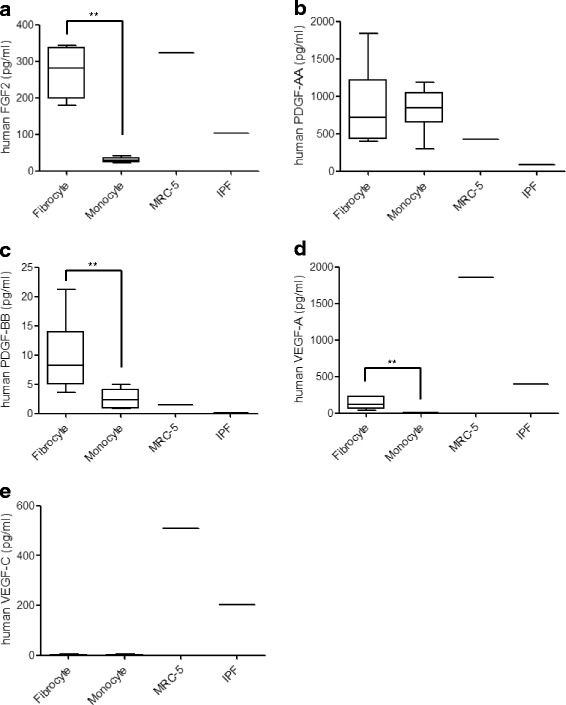
Production of growth factors from fibrocytes, monocytes, and fibroblasts. **a** FGF2, **b** PDGF-AA, **c** PDGF-BB, **d** VEGF-A, and **e** VEGF-C were measured in the cell culture supernatants of fibrocytes from three different donors (1–3), monocytes from three different donors (1–3), and human normal fibroblasts (MRC-5 and IPF-fibroblasts) using ELISA. Data were analyzed by the Mann–Whitney *U* test and are displayed as median and interquartile range of six samples (fibrocyte and monocyte) and each cell line (MRC-5 and IPF cell). In all graphs: **P < 0.01

**Fig. 2:**
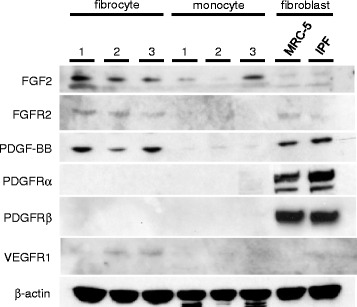
Expression of growth factor receptors on fibrocytes, monocytes, and fibroblasts. The expression of growth factors (FGF2 and PDGF-BB) and their receptors (FGFR2, PDGFRα, PDGFRβ, and VEGFR1) was measured in cell extracts of fibrocytes from three different donors (1–3), monocytes from three different donors (1–3), and human lung fibroblasts (MRC-5 and IPF-fibroblasts) by an immunoblot analysis

We confirmed the expression of growth factors in fibrocytes as previously reported [18]. In the present study, we compared their expression among monocytes, fibrocytes, and fibroblasts. Based on the targets of nintedanib, FGF2, PDGF-AA, PDGF-BB, VEGF-A, VEGF-B, VEGF-C, and TGFβ-1 were examined in the different culture supernatants using ELISA. Fibrocytes secreted greater amounts of FGF2, PDGF-BB, and VEGF-A than monocytes (Fig. 1a–d). Fibrocytes and fibroblasts both secreted PDGF-AA (Fig. 1b). Only fibroblasts secreted VEGF-C (Fig. 1e). PDGF-AB, TGFβ-1, and VEGF-B were below the detection limit of ELISA. The expression of FGF2 and PDGF-BB from fibrocytes was also confirmed by an immunoblot analysis (Fig. 2). These results suggest that fibrocytes are one of the sources of growth factors in pulmonary fibrosis.

### Fibrocytes and fibroblasts express growth factor receptors, which are the targets of nintedanib

The expression of growth factor receptors on fibrocytes, monocytes, and fibroblasts was examined by an immunoblot analysis. Fibrocytes expressed FGFR2 and VEGFR1. Fibroblasts also expressed FGFR2, and strongly expressed PDGFRα and β (Fig. 2).

### Nintedanib inhibits the proliferation of lung fibroblasts induced by fibrocytes by blocking the phosphorylation of growth factor receptors on fibroblasts

**Fig. 3:**
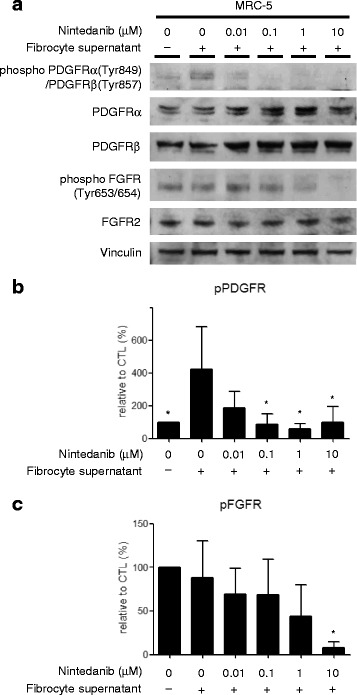
Nintedanib inhibits the phosphorylation of growth factor receptors on fibroblasts induced by fibrocytes. Human lung fibroblasts (MRC-5) were incubated with nintedanib at different concentrations and with the cell culture supernatant of fibrocytes. The expression of all receptors and receptor phosphorylation were measured by an immunoblot analysis. Representative immunoblot (**a**) and corresponding densitometric quantification of PDGFR phosphorylation (**b**; *n* = 3 independent experiments) and FGFR phosphorylation (**c**; n = 3 independent experiments). Data were analyzed using a one-way ANOVA and are displayed as means ± SDs in three separate experiments. For all graphs: *P < 0.05 versus the value in the group treated with fibrocyte supernatants alone

In order to examine the effects of culture supernatants of fibrocytes as well as those of nintedanib on the phosphorylation of growth factor receptors, the expression of all receptors and receptor phosphorylation were examined using an immunoblot analysis. The incubation of MRC-5 cells with the culture supernatant of fibrocytes resulted in the phosphorylation of PDGFR, which was inhibited by nintedanib mainly at a concentration of 100 nM or more. However, the inhibitory effects of nintedanib were more potent on the phosphorylation of PDGFR compared to FGFR (Fig. 3a–c). These results indicate that growth factors produced by fibrocytes have a biological activity to stimulate fibroblasts, which can be inhibited by nintedanib.

**Fig. 4:**
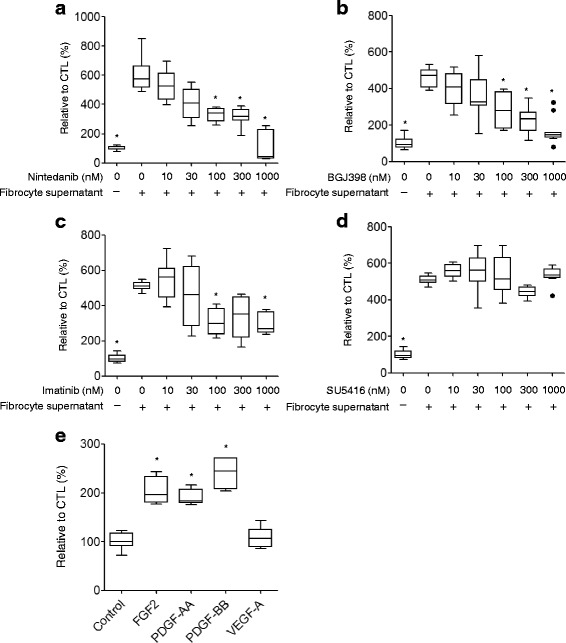
Nintedanib inhibits the growth of fibroblasts in response to growth factors produced by fibrocytes. The proliferation of fibroblasts cultured in the cell culture supernatant of fibrocytes with various concentrations of **a** nintedanib, **b** BGJ398, **c** imatinib, and **d** SU5416 (0–1 μM) was measured by a ^3^H thymidine incorporation assay. The proliferation of fibroblasts cultured with recombinant growth factors (FGF2: 30 ng/ml, PDGF-AA: 100 ng/ml, PDGF-BB: 100 ng/ml, VEGF-A: 100 ng/ml) for 72 h. 3H–TdR was pulsed for the final 18 h, and the incorporation of 3H–TdR was measured. **e** Data were analyzed using the Kruskal-Wallis H test and are displayed as median and interquartile range (*n* = 8–11, independent experiments each). In all graphs: *P < 0.05 versus the value in the group treated with fibrocyte supernatants alone **a**-**d** or control **e**

Next, we investigated the effects of nintedanib on the fibrocyte-induced proliferation of lung fibroblasts. Fibroblast proliferation induced by the culture supernatant of fibrocytes was inhibited by nintedanib mainly at a concentration of 100 nM or more (Fig. 4a). The selective inhibition of FGFR and PDGFR by their specific inhibitors, BGJ-398 and imatinib, respectively, also inhibited fibroblast proliferation (Fig. 4b, c), whereas the VEGFR inhibitor SU5416 did not (Fig. 4d). These results were supported by recombinant FGF2, PDGF-AA, and PDGF-BB also stimulating the proliferation of fibroblasts, whereas VEGF-A did not (Fig. 4e). Taken together, these results show that FGF and PDGF, but not VEGF are important for the fibrocyte-mediated proliferation of fibroblasts.

### Nintedanib inhibits the differentiation of fibrocytes from HMNC

We examined the effects of nintedanib on the differentiation of fibrocytes. We used human fibrocytes generated from HMNC by culturing them on fibronectin-coated 6-well plates for six days.

Various kinase inhibitors, including nintedanib, BGJ398, imatinib, SU5416, and SB431542, in addition to serum amyloid P were added to the culture medium of human HMNC at the concentrations ranging from 0 to 1 μM (Additional file 2: Figure S1). As reported previously, serum amyloid P inhibited the differentiation of fibrocytes [21]. Specific inhibitors including nintedanib, BGJ398, imatinib, and SU5416 reduced the number of fibrocytes generated by HMNC, whereas SB431542, which is a kinase inhibitor for the receptor of TGFβ, did not.

**Fig. 5:**
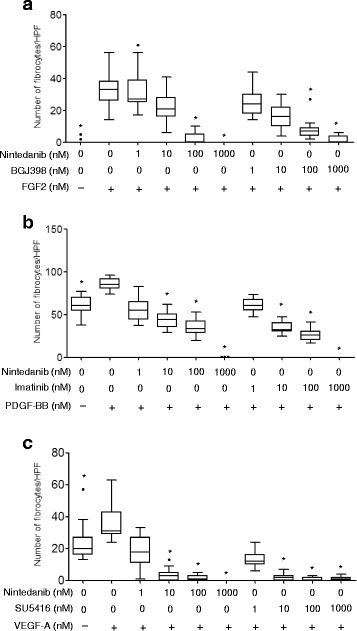
Nintedanib inhibits the differentiation of fibrocytes induced by growth factors. HMNC were cultured on fibronectin-coated dishes in medium containing low amounts of FBS. Recombinant growth factors such as (**a**) FGF-2 (30 ng/ml), (**b**) PDGF-BB (100 ng/ml), and (**c**) VEGF-A (100 ng/ml) and growth factor inhibitors (BGJ398 for FGFR, imatinib for PDGFR, SU5416 for VEGFR, and nintedanib) were administrated every 48 h. On day 6, attached cells were stained with the Diff-quick stain, and counted in 5 fields at 100× magnification. Data were analyzed using the Kruskal-Wallis H test and are displayed as median and interquartile range of three separate experiments (*n* = 15 in each group). In all graphs: *P < 0.05 versus the group treated with growth factors (FGF2 or PDGF-BB or VEGF-A) alone. HPF; high-power fields

In order to confirm that these results were due to the activation of growth factor receptors, we investigated whether the addition of the recombinant proteins of growth factors increased the generation of fibrocytes from HMNC. Recombinant FGF2, PDGF-BB, and VEGF-A increased the number of fibrocytes generated by HMNC (Fig. 5a, b, and c). In contrast, BGJ398, imatinib, and SU5416 inhibited growth factor-induced differentiation corresponding to each specific inhibitor (Fig. 5a, b, and c). Nintedanib with the concentration of 100 nM or more also inhibited differentiation promoted by all growth factors (Fig. 5a, b, and c).

In an attempt to rule out the possibility that these results were due to the cytotoxic activity of nintedanib against fibrocytes, we investigated the effects of nintedanib on the viability of human fibrocytes derived from monocytes in vitro. Nintedanib was added to a culture of fibrocytes on day 6. The treatment of fibrocytes with nintedanib at concentrations up to 1 μM for six days did not decrease the number of fibrocytes harvested on day 12 (Additional file 2: Figure S2), indicating that the cellular toxicity of nintedanib on fibrocytes was negligible.

### Nintedanib inhibits the migration of fibrocytes induced by growth factors

**Fig. 6:**
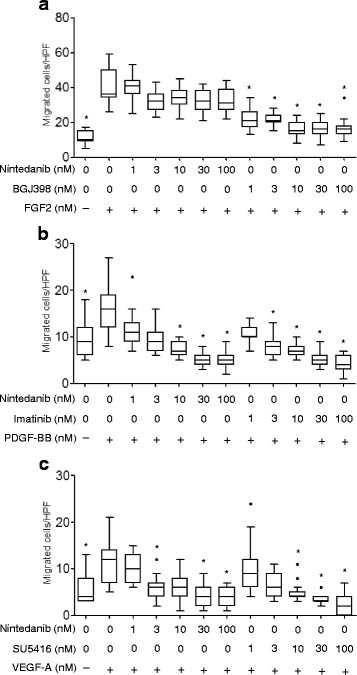
Nintedanib inhibits the migration of fibrocytes induced by growth factors. Fibrocytes were added to the upper chamber in the presence or absence of various concentrations of nintedanib (**a**, **b**), BGJ398 (**a**), imatinib (**b**), or SU5416 (**c**). A total of 30 ng/ml of FGF2 (**a**), 100 ng/ml of PDGF-BB (**b**), or 100 ng/ml of VEGF-A (**c**) was added to the lower chamber. After a 20-h incubation, fibrocytes that had migrated to the bottom surface of the filter were counted in 5 random fields per section at 100× magnification. Data were analyzed using the Kruskal-Wallis H test and displayed as median and interquartile range of three separate experiments (n = 15 in each group). In all graphs: *P < 0.05 versus the group treated with growth factors (FGF2, PDGF-BB, or VEGF-A) alone. HPF; high-power fields

We assessed the effects of kinase inhibitors on the migration of human fibrocytes induced by various growth factors at concentrations up to 100 nM. The number of migrated fibrocytes markedly increased when cells were treated with FGF2, PDGF-BB or VEGF-A. Moreover, BGJ398, imatinib, and SU5416 inhibited migration mediated by growth factors corresponding to each inhibitor (Fig. 6a, b, and c). Nintedanib with the concentration of 30 nM or more also inhibited differentiation promoted by PDGF-BB and VEGF-A, but didn’t inhibited differentiation promoted by FGF2 (Fig. 6a).

### Nintedanib reduces the number of fibrocytes accumulating in the mouse lung with BLM-induced pulmonary fibrosis

**Fig. 7:**
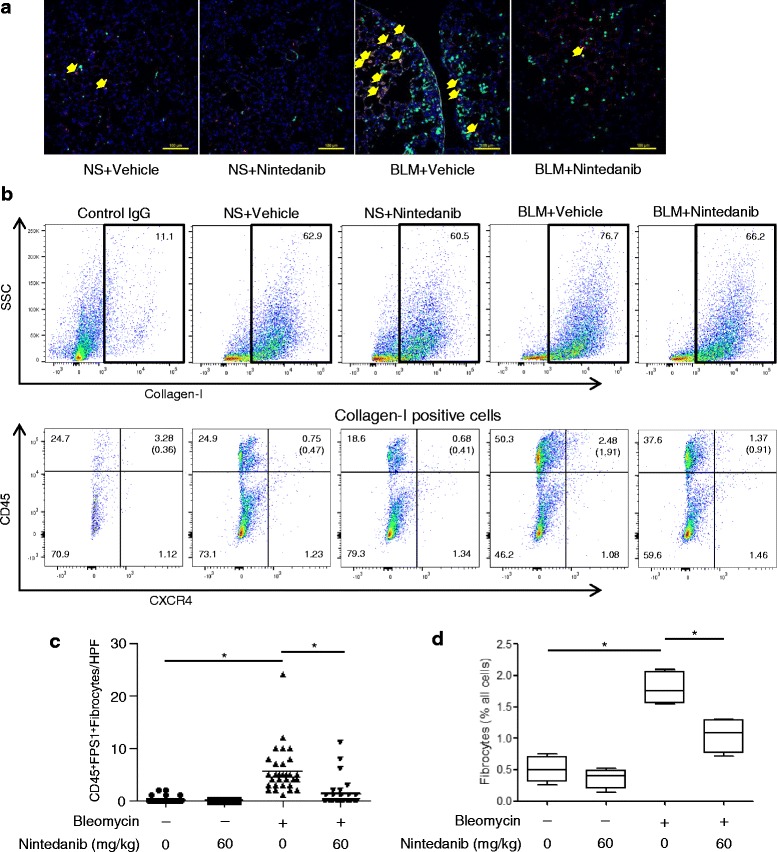
Nintedanib regulates the pool size of fibrocytes in bleomycin (BLM)-treated mouse lungs. Normal saline (NS) or BLM-treated mice with or without nintedanib (60 mg/kg/day) were killed on day 7. Paraffin-embedded lung sections were stained with a rabbit anti-S100A4/FSP-1 antibody (green) and anti-CD45 (red), and lung digests stained for collagen-1, CD45, and CXCR4 were examined by flow cytometry. Lung digests were also stained for isotype control IgG labeled with FITC, phycorythrin, or phycoerythrin-cyanine. **a** Representative images of immunohistochemical staining in each group are shown. Arrows indicate fibrocytes doubled stained for S100A4/FSP-1 and CD45 (scale bar = 100 μm). **b** After lung digests were first gated by collagen-1 as shown in above figures, only collagen-1+ cells were examined for dual expression of CD45 and CXCR4 using the logical gates depicted as shown in below figures. Collagen-1^+^ cells in lung digests were examined for the dual expression of CD45 and CXCR4 using the logical gates depicted. Collagen-1^+^CD45^+^CXCR4^+^ fibrocytes in lung digests from mice in each group were examined. **c** S100A4/FSP-1 and CD45 double-positive cells were counted in 10 random fields per section at 20× magnification for three separate lung section. Data were analyzed using a one-way ANOVA and displayed as dot plot and their means (*n* = 30 in each group). **d** The percentage of fibrocytes relative to all cells in lung digests were examined. Data were analyzed using the Kruskal-Wallis H test and displayed as median and interquartile range of four separate experiments. In all graphs: *P < 0.01 versus the group treated with BLM alone

We investigated whether nintedanib reduces the number of fibrocytes in a mouse model of BLM-induced pulmonary fibrosis. Mice received a single transbronchial instillation of BLM on day 0. After the administration of nintedanib with the concentration of 60 mg/kg by gavage from days 0 to 7, lung tissues were harvested and analyzed. Paraffin-embedded lung sections were stained for S100A4/fibroblast-specific antigen-1 (FSP-1) and CD45. Because FSP-1 is expressed not only by fibrocytes but also by fibroblasts and endothelial cells, we counted only strongly stained cells as FSP-1 positive cells. Double-positive cells for S100A4/FSP-1 and CD45 in lung sections were defined as fibrocytes as described previously [17] (Fig. 7a). The administration of BLM significantly increased the number of fibrocytes per high-power field in the lungs of BLM-treated mice, while nintedanib at 60 mg/kg significantly reduced these numbers (Fig. 7c). We also counted the number of fibrocytes in the lungs of BLM-treated mice using a three-color FACS analysis (CXCR4, collagen-1, and CD45). The percentage of fibrocytes (CXCR4^+^Col1^+^CD45^+^cells) among all lung cells in BLM-treated mice was elevated on day 7, and nintedanib at 60 mg/kg was found to significantly reduce this percentage (Fig. 7b, d). Consequently, when nintedanib was continuously administrated until day 21, the number of fibrotic lesions in the lungs of BLM-treated mice was reduced (Additional file 2: Figure S3). A quantitative histological analysis showed that the Ashcroft fibrotic score was significantly lower in mice treated with BLM and nintedanib at a dose of 60 mg/kg than in those treated with BLM alone (Additional file 2: Figure S4A). A hydroxyproline colorimetric assay also showed reductions in collagen content in the lungs of mice treated with nintedanib at 60 mg/kg (Additional file 2: Figure S4B). The anti-fibrotic effects of nintedanib were consistent with those described previously [7].

## Discussion

Nintedanib is a tyrosine kinase inhibitor that was recently approved for the treatment of patients with IPF in the major parts of the world. However, the mechanisms by which nintedanib attenuates pulmonary fibrosis have not been fully clarified. In the present study, we examined the effects of nintedanib on fibrocytes in order to improve understanding of the anti-fibrotic effects of nintedanib. Nintedanib inhibited the migration and differentiation of fibrocytes in vitro. The activity of fibrocytes to stimulate the proliferation of fibroblasts was also blocked by nintedanib. In addition, treatment with nintedanib significantly reduced the accumulation of fibrocytes in the lungs of a BLM-induced pulmonary fibrosis model in mice.

Fibrocytes are monocyte-derived cells that are regarded as a subpopulation of mesenchymal progenitor cells, and express the markers of hematopoietic cells (CD34), leukocytes (CD11b, CD13, and CD45), and fibroblast products (collagens I and III and fibronectin) [12]. Fibrocytes have been implicated in the pathogenesis of pulmonary fibrosis [22, 23]. Since fibrosis is characterized by the accumulation of activated fibroblasts and excessive deposition of fibrotic extracellular matrix proteins including type I collagen, fibrocytes have been proposed as an important direct contributor to pulmonary fibrosis. However, a recent study demonstrated the negligible role of fibrocytes in the production of collagen in a BLM-induced pulmonary fibrosis model [24]. On the other hand, we showed that fibrocytes are a cluster of cells that produce various growth factors including FGF2, PDGF-BB, and VEGF-A [18]. These findings indicate that fibrocytes are an important cell population responsible for the production of ligands in signaling pathways that are targeted by nintedanib.

Nintedanib is a potent tyrosine kinase inhibitor that targets FGFR, PDGFR, and VEGFR [7–9]. In the present study, we showed that fibrocytes expressed FGFR2 and VEGFR1 using an immunoblot analysis. We previously demonstrated the expression of PDGFRα and β using qPCR and flow cytometric analyses [17]; however, the detection of PDGFRα and β by immunoblot analysis was not sufficient in the present study. This may have been due to the method used to detect the expression of PDGFR, as PDGF proteins may stimulate the migration of fibrocytes [17]. We also showed that fibroblasts express FGFR2 and PDGFRα and β. Hence, fibrocytes and fibroblasts may be putative therapeutic target cells for nintedanib.

The supernatant of fibrocytes stimulated the phosphorylation of tyrosine kinase receptors on fibroblasts, and the inhibitory effects of nintedanib on these receptors were demonstrated. Despite PDGFR of fibroblasts was phosphorylated by addition of the culture supernatant of fibrocytes, FGFR had been phosphorylated regardless whether the fibrocyte supernatant was added or not. Furthermore, although the inhibitory effects of nintedanib on the phosphorylation of PDGFR were observed at approximately 10–100 nM, the inhibition of FGFR phosphorylation required 100–1000 nM. These differences might be due to the two reason. First, fibroblasts also produce FGF2, as shown in Fig. 1 and/or due to the differences in the inhibitory potency. Second, The IC_50_ values of nintedanib for PDGFRα and PDGFRβ have been reported to be in the range of 41–58 nM, whereas that for FGFR2 is 257 nM [7].

The activation of receptors on fibroblasts induced by fibrocyte supernatant resulted in their proliferation. Nintedanib and BGJ398, the FGFR inhibitor, attenuated this proliferation at concentrations rather than 100 nM. Although imatinib, a PDGFR inhibitor, also suppressed the growth of fibroblasts, it required concentrations greater than 1 μM. The IC_50_ value of imatinib against PDGFR was reported to be in the range of 100 nM to 380 nM [25]. A direct comparison between imatinib and nintedanib is difficult due to the different cell types used, but the IC_50_ value of imatinib was considered to be higher than that of nintedanib in the present study. SU5416, a VEGF inhibitor, did not exert inhibitory effects against cell growth, even when 1 μM was used as the maximum concentration. Since the production of VEGF by fibrocytes was less than that by fibroblasts, as shown in Fig. 1, FGF and PDGF are considered to be more important than VEGF as growth factors produced by fibrocytes that activate the proliferation of fibroblasts.

We also investigated the effects of nintedanib on the differentiation of fibrocytes from monocytes. In the present study, inhibitory effects were observed not only by nintedanib, but also by specific inhibitors for FGFR, PDGFR, and VEGFR. We also demonstrated that several growth factors including PDGF, FGF, and VEGF stimulated the differentiation of fibrocytes. The differentiation of fibrocytes is reported to be augmented by fibrogenic cytokines such as interleukin (IL)-4 and IL-13 along with PDGF [26]. However, the relationship between the differentiation of fibrocytes and the FGF/FGFR or VEGF/VEGFR signaling pathways has not been examined. Monocytes have been shown to express FGFR [27], PDGFR [28], and VEGFR [29], however growth factor receptors were not clearly detected by immunoblot on monocytes in the present study as shown in Fig. 2. Therefore, these growth factors may play a role in the differentiation of fibrocytes in pulmonary fibrosis.

We showed that nintedanib inhibited the migration of fibrocytes. In our previous study, the PDGF/PDGFR axis was found to play a role in the migration of fibrocytes into fibrotic lungs in vitro and in vivo [17]. Furthermore, we demonstrated that FGF and VEGF were potent chemoattractants for fibrocytes in the present study. Therefore, nintedanib is considered to prevent pulmonary fibrosis by directly inhibiting the differentiation and migration of fibrocytes. However, nintedanib could not inhibit the migration of fibrocytes stimulated by FGF2. As mentioned above, it is considered that this is because the IC_50_ value of nintedanib against FGFR is high. The effects of nintedanib in in vitro experiments were also confirmed in in vivo experiments. The administration of nintedanib significantly reduced the number of fibrocytes in the lung tissues of mice treated with BLM to induce pulmonary fibrosis.

The limitation of this study was that it was difficult to examine the effect of nintedanib on fibrocyte induction in the fibrotic phase in our model. Because in bleomycin-induced pulmonary fibrosis model in mice, the number of fibrocytes induced to the lung begins to increase from day 7, but drastically end up decreasing on day 21 as shown in previous report [17]. Therefore, to circumvent this problem, the analysis of lung tissue from patients who had nintedanib may be more informative to see the impact on fibrocyte recruitment in future study.

## Conclusions

In summary, the present study clearly demonstrated a novel anti-fibrotic activity of nintedanib. Nintedanib directly inhibited the migration and differentiation of fibrocytes. In addition, nintedanib blocked the receptors of pro-fibrotic growth factors which are stimulated by mediators produced by fibrocytes (Additional file 2: Figure S5).

## Additional files

Additional file 1: Detailed methods, and figure legends of Figure S1-S5. (PDF 227 kb)

Additional file 2: **Figure S1**. Nintedanib inhibits the differentiation of fibrocytes generated from HMNC. **Figure S2**. Nintedanib did not cause cellular damage in fibrocytes. **Figure S3**. Histological examination of the anti-fibrotic effects of nintedanib on bleomycin (BLM)-induced lung fibrosis. **Figure S4**. Quantitative examination of the anti-fibrotic effects of nintedanib on bleomycin (BLM)-induced pulmonary fibrosis. **Figure S5**. Anti-fibrotic role of nintedanib in pulmonary fibrosis via the suppression of fibrocyte activity. (PDF 884 kb)

## Abbreviations

^3^H–TdR: [^3^H] thymidine deoxyribose
BLM: Bleomycin
ELISA: Enzyme-linked immunosorbent assay
FACS: Fluorescence-activated cell sorter
FGF: Fibroblast growth factor
FSP-1: Fibroblast-specific antigen-1
HMNC: Human mononuclear cells
IL: Interleukin
IPF: Idiopathic pulmonary fibrosis
NS: Normal saline
PDGF: Platelet-derived growth factor
TGF: Transforming growth factor
VEGF: Vascular endothelial growth factor
