# Supplementary material for: Anti-fibrotic efficacy of nintedanib in pulmonary fibrosis via the inhibition of fibrocyte activity
Source: Respir Res. 2017 Sep 15;18:172. doi: 10.1186/s12931-017-0654-2 (PMC5603061; doi:10.1186/s12931-017-0654-2)
Supplement: Supplementary file 1 — Detailed methods, and figure legends of Figure S1-S5. (PDF 227 kb) [file 12931_2017_654_MOESM1_ESM.pdf]

Anti-fibrotic efficacy of nintedanib in pulmonary fibrosis via the inhibition fibrocyte activity

Seidai Sato[1], Shintaro Shinohara[1], Shinya Hayashi[1], Shun Morizumi[1], Shuichi Abe[1], Hiroyasu Okazaki[1], Yanjuan Chen[1], Hisatsugu Goto[1], Yoshinori Aono[1, 2], Hirohisa Ogawa[3], Kazuya Koyama[1], Haruka Nishimura[1], Hiroshi Kawano[1], Yuko Toyoda[1], Hisanori Uehara[3], Yasuhiko Nishioka[1]

SUPPLEMENT DATA

Materials and Methods

Isolation of human fibrocytes and monocytes

Human fibrocytes were isolated according to a previously described method (E1, E2). All procedures for consent, sample collection, and privacy protection were approved by the Ethics Committee of Tokushima University Hospital. Human mononuclear cells (HMNC) were isolated from the peripheral blood of healthy volunteers using Ficoll density centrifugation (E3). Isolated cells were cultured in DMEM supplemented with 20% fetal bovine serum (FBS), penicillin (100 U/ml), and streptomycin (50 μg/ml) on bovine fibronectin (R&D systems, Minneapolis, MN)-coated 150-mm cell culture dishes (BD Pharmingen, Franklin Lakes, NJ). After six to seven days, medium was

aspirated and washed with sterile phosphate-buffered saline (PBS) at least six times to remove floating cells. Adherent cells were defined as circulating fibrocyte-like cells using a flow cytometric analysis and immunostaining. Monocytes were isolated from HMNC with CD14 microbead antibodies and an automated magnetic cell separation device (Automacs^{TM} Pro separator; Miltenyi Biotec K.K. Singapore). Informed consent was obtained from all volunteers.

Materials

Nintedanib and SB431542 were obtained from Boehringer Ingelheim GmbH & Co. KG (Biberach, Germany). SU5416, a VEGFR-specific inhibitor, was purchased from Abcam (Cambridge, MA). BGJ-398 and imatinib were purchased from Chemietek (Indianapolis, IN, USA). Bleomycin (BLM) was purchased from Nippon Kayaku Co. (Tokyo, Japan). Recombinant FGF2 was purchased from PeproTech (Rocky Hill, NJ). Recombinant PDGF-BB was purchased from Abcam (Cambridge, UK). Recombinant VEGF-A was purchased from R&D Systems (Minneapolis, MN). Rabbit anti-S100A4/FSP-1 antibody was purchased from Thermofisher Scientific (Waltham, MA). Rat anti-CD45 antibody was purchased from BD Pharmingen (Franklin Lakes, NJ).

Cell lines

The human lung fibroblast cell line, MRC-5 was purchased from DS PHARMA BIOMEDICAL (Osaka, Japan). IPF-fibroblasts (IPF) were from a primary culture of lung tissues from patients with IPF. Cells were maintained in DMEM medium supplemented with 10% FBS, penicillin (100 U/ml), and streptomycin (50 μg/ml). All cells were cultured at 37°C in a humidified atmosphere of 5% CO_2 in air.

Cell culture supernatants

Fibrocytes, monocytes, MRC-5 cells, and IPF-fibroblasts (1×10^6 cells/3 ml) were cultured in 6-well plates with 0.1% FBS DMEM for 24 hours, and cell culture supernatants were collected.

Measurement of growth factors

Mediator concentrations were measured in the cell culture supernatants of fibrocytes, monocytes, and fibroblasts (MRC-5 cells and IPF-fibroblasts) with commercial enzyme-linked immunosorbent assay (ELISA) kits. ELISA kits and their sensitivities were as follows: FGF2, PDGF-AA, PDGF-BB, PDGF-AB, and TGF beta 1 (Abcam, Cambridge, UK), with sensitivities of 50, 2.5, 1, 10 and 80 pg/ml, respectively; VEGF-A (eBioscience, San Diego, CA) with a sensitivity of 7.9 pg/ml; VEGF-B (Abnova, Jhouzih St, Taipei, Taiwan) with a sensitivity of 8 pg/ml; VEGF-C (R&D Systems, Minneapolis, MN), with a sensitivity of 4 pg/ml.

Immunoblot analysis

Whole cell extracts were prepared with M-PER reagents (Thermo Fisher Scientific, Waltham, MA USA) containing phosphatase and protease inhibitor cocktails (Roche, Basel, Switzerland).

In order to compare the expression of the receptors and growth factors in each cell, cell extracts of fibrocytes and monocytes from three different donors were collected shortly after their isolation. Cell extracts from MRC-5 and IPF-fibroblasts were collected after culturing in DMEM containing 10% FBS.

In order to examine the effects of growth factors produced by fibrocytes and nintedanib on the phosphorylation of growth factor receptors, MRC-5 cells were cultured in culture medium containing FBS 0.1%, and were then incubated with nintedanib at different concentrations (0-10 μM) or the cell culture supernatant of fibrocytes for 10 minutes. Cell extracts were then collected.

Protein concentrations were measured using the Bradford method. The same amounts of total cell extract proteins were electrophoresed on 4-10% NuPAGE Bis-Tris Mini Gels. Gel proteins were then electrphoretically transferred onto polyvinylidene difluoride membranes (Millipore, Billerica, MA) using the WSE-4040 HorizeBLOT 4M-R system (ATTO, Tokyo, Japan). The membrane was treated with the blocking agent Blocking One (Nacalai Tesque) for one hour and incubated at 4°C overnight with

the first antibodies. Following 4 washes, the membrane was incubated with horseradish peroxidase-conjugated secondary antibodies (GE Healthcare, Fairfield, CT) in buffer at room temperature for one hour. The membrane was washed and developed using Amersham ECL Western Blotting Detection Reagents (GE Healthcare, Fairfield, CT), and signals were detected using an enhanced chemiluminescence system (GE Healthcare, Fairfield, CT).

The first antibodies used were as follows: anti-FGF2 antibody (1:1000 dilution, ab106245, Abcam, Cambridge, UK), anti-FGF Receptor 2 antibody (1:1000 dilution, #11835, Cell Signaling Technology, Danvers, MA), anti-phospho FGF Receptor antibody (1:1000 dilution, #3471, Cell Signaling Technology, Danvers, MA), anti-PDGF-BB antibody (1:000 dilution, ab23914, Abcam, Cambridge, UK), anti-PDGF Receptor α antibody (1:1000 dilution, #3164, Cell Signaling Technology, Danvers, MA), anti- PDGF Receptor β antibody (1:1000 dilution, #3169, Cell Signaling Technology, Danvers, MA), anti-Phospho-PDGF Receptor α/β antibody (1:1000 dilution, #3170, Cell Signaling Technology, Danvers, MA), anti-Vinculin antibody (1:1000 dilution, #4650, Cell Signaling Technology, Danvers, MA), and anti-β-actin antibody (1:200 dilution, Santa Cruz, Dallas, TX). Densitometric quantification was done by Image J 1.6.0_24 software (National Institute of Health).

Proliferation assay

MRC-5 cells (8 x 10^3 cells/well) were seeded on a 96-well plate, and cultured in the cell culture supernatant of fibrocytes with various concentrations of nintedanib, BGJ398, imatinib, and SU5416 (0-1 μM) or in medium containing recombinant growth factors (FGF2: 30 ng/ml, PDGF-AA: 100 ng/ml, PDGF-BB: 100 ng/ml, VEGF-A: 100 ng/ml) for 72 hours. One μCi/well of [^{3}H] thymidine deoxyribose (^{3}H-TdR) was pulsed for the final 18 hours and the incorporation of ^{3}H-TdR was measured using a liquid scintillation counter (E1).

Differentiation assay with inhibitors

HMNC (200 x 10^4cells/ml) were seeded in fibronectin-coated 6-well plates with DMEM containing 20% FBS and inhibitors. Various concentrations of inhibitors such as nintdanib, BGJ398, imatinib, SU5416, and SB431542 (0-1 μM) were added every 72 hours. Serum amyloid P (0-1 μM) was added every 48 hours as reported previously (E4). On day 6, floating cells were washed out with PBS more than six times, and adherent cells were trypsinized and counted as human fibrocytes. All experiments were performed in triplicate.

Differentiation assay with recombinant growth factors

HMNC (200 x 10^4cells/ml) were seeded in fibronectin-coated 6-well plates with DMEM containing 4% FBS, growth factors (FGF2: 30 ng/ml, PDGF-BB: 100 ng/ml,

VEGF-A: 100 ng/ml), and various concentrations of inhibitors (nintedanib, BGJ398, imatinib, and SU5416: 0-1 μM). Every growth factor and inhibitor was added again every 48 hours. On day 6, attached cells were stained with Diff-Quick (Baxter, Miami, FL), and counted in five fields at 100x magnification. All experiments were performed in triplicate.

Cytotoxicity of nintedanib against fibrocytes

HMNC (200 x 10^4cells/ml) were cultured on fibronectin-coated 6-well plates with DMEM containing 20% FBS from days 0 to 6. On day 6, after floating cells were washed out with PBS more than six times, various concentrations of nintedanib (0-1 μM) were added with new DMEM. Nintedanib was added on day 9 again, and the number of adherent cells was counted on day 13 after trypsinization. All experiments were performed in triplicate.

Cell migration assay

The migration assay was performed using cell culture inserts with a pore size of 8 μm (BD Bioscience, San Jose, CA, USA) (E1, E2). Fibrocytes in DMEM containing 0.1% of FBS were added to the upper chamber in the presence or absence of various concentrations of nintedanib, BGJ398, or imatinib (0-100 nM). A total of 30 ng/ml of FGF2 or 100 ng/ml of PDGF-BB or 100 ng/ml of VEGF-A was added to the lower

chamber. After a 20 hour incubation, fibrocytes that had migrated to the bottom surface of the filter were stained with Diff-Quick (Baxter, Miami, FL), and counted in five randomly selected fields on each filter under a microscope at 200x magnification. All experiments were performed in triplicate.

BLM-induced lung fibrosis in mice

Eight-week-old C57BL/6 male mice were purchased from CLEA Japan (Tokyo, Japan). Mice were maintained in the animal facility of Tokushima University under specific pathogen-free conditions according to the guidelines of our university (E3). The present study was approved by the Institutional Animal Care and Use Committee of Tokushima University (Permission Number: 14099).

Eight-week-old C57BL/6 mice received a single transbronchial installation of 7.5 mg/kg NaCl or BLM on day 0. Nintedanib or distilled water was administered daily by gavage at 30 and 60 mg/kg from days 0 to 7 or 21. The lung tissues analyzed on day 7 were used in a FACS analysis and immunohistochemistry. The lung tissues analyzed on day 21 were used for a histopathological analysis and in a hydroxyproline colorimetric assay.

Histopathology

Right lung tissues were harvested, fixed in 10% formalin, and embedded in paraffin.

Three-micrometer-thick sections were stained with hematoxylin and eosin (H&E) or azan mallory. In the quantitative analysis, a numeric fibrotic scale was used (Ashcroft score) (E5). The mean score was considered to be the fibrotic score.

Immunohistochemistry

Paraffin-embedded lung sections were stained with primary antibodies (rabbit anti-S100A4/FSP-1 antibody and rat-anti-CD45 antibody) at 4°C overnight and subsequently stained with fluorescence-conjugated secondary antibodies and 4’, 6-diamidino-2-phenylindole at room temperature for 1 hour (E1). Fluorescence images were captured with a confocal laser scanning microscope at 20x magnification (Nikon A1R resonant scanning confocal system, Tokyo, Japan). Double-positive cells for S100A4/FSP-1 and CD45 were counted in five randomly selected fields by using three independent lung sections.

FACS

Minced lungs were digested, and harvested cells were stained with phycoerythrin-cyanine 5-labeled anti-mouse CD45 antibody, phycoerhthrin-labeled anti-mouse CXCR4 antibody, and biotin-conjugated anti-mouse collagen-1 antibody, followed by streptavidin-FITC. Stained cells were analyzed using a FACScan flow cytometer (BD Biosciences-Pharmingen, San Diego, CA) (E1).

Statistical analysis

The significance of differences was analyzed using a Mann–Whitney *U* test for unpaired samples or a one-way ANOVA followed by a Dunnett's test. Where appropriate, the Kruskal-Wallis H test was applied with Dunn’s test. *P* values of less than 0.05 were considered to be significant. Statistical analyses were performed using GraphPad Prism programme Ver. 5.01 (*GraphPad* Software Inc.).

References

E1. Aono Y, Kishi M, Yokota Y, Azuma M, Kinoshita K, Takezaki A, Sato S, Kawano H, Kishi J, Goto H, Uehara H, Izumi K, Nishioka Y. Role of platelet-derived growth factor/platelet-derived growth factor receptor axis in the trafficking of circulating fibrocytes in pulmonary fibrosis. Am J Respir Cell Mol Biol. 2014; 51:793-801.

E2. Makino H, Aono Y, Azuma M, Kishi M, Yokota Y, Kinoshita K, Takezaki A, Kishi J, Kawano H, Ogawa H, Uehara H, Izumi K, Sone S, Nishioka Y. Antifibrotic effects of CXCR4 antagonist in bleomycin-induced pulmonary fibrosis in mice. J Med Invest. 2013;60:127-37.

E3. Nishioka Y, Nishimura N, Suzuki Y, Sone S. Human monocyte-derived and CD83(+) blood dendritic cells enhance NK cell-mediated cytotoxicity. Eur J Immunol. 2001; 31:2633-2641.

E4. Pilling D, Buckley CD, Salmon M, Gomer RH. Inhibition of fibrocyte differentiation by serum amyloid P. J Immunol. 2003 15;171:5537-5546.
E5. Ashcroft T, Simpson JM, Timbrell V. Simple method of estimating severity of pulmonary fibrosis on a numerical scale. J Clin Pathol. 1988; 41:467-70.

Figure legends

Figure S1.

Nintedanib inhibits the differentiation of fibrocytes. HMNC were cultured for six days on fibronectin-coated dishes with kinase inhibitors in DMEM containing 20% FBS. Inhibitors such as (A) nintedanib, (B) BGJ398, (C) imatinib, (D) SU5416, and (F) SB431542 were added every 72 hours. (E) Serum amyloid P was added every 48 hours. On day 6, the number of adherent cells was counted as human fibrocytes. (G) Representative photographs of fibrocytes generated from HMNC treated with nintedanib on day 6 (Scale bar = 100μm). Data were analyzed using the Kruskal-Wallis H test and displayed as median and interquartile range of six separate experiments. In all graphs: *P<0.05 versus the group treated without each inhibitor.

Figure S2

Nintedanib did not cause cellular damage in fibrocytes. After HMNC were cultured on fibronectin-coated dishes for six days, nintedanib was added on days 6 and 9. On day 12,

the number of adherent cells was counted. (A) Data were displayed as median and interquartile range of four separate experiments. (B) Representative photographs of fibrocytes treated with nintedanib on day 12 (Scale bar = 100μm).

Figure S3.

Histological examination of the anti-fibrotic effects of nintedanib on bleomycin (BLM)-induced lung fibrosis. C57BL/6 mice received an intranasal instillation of 7.5 mg/kg NaCl or BLM. Nintedanib was administered each day by gavage at 30 and 60 mg/kg from days 0 to 21. Analyses were performed on day 21. A histological examination was performed by staining with hematoxylin and eosin (H&E) (A, C, E, G, and I) and azan mallory (B, D, F, H, and J) (scale bar = 1mm). (A, B) Distilled water. (C, D) nintedanib (60 mg/kg) alone. (E, F) BLM alone. (G, H) BLM + nintedanib (30 mg/kg). (I, J) BLM + nintedanib (60 mg/kg).

Figure S4.

Quantitative examination of the anti-fibrotic effects of nintedanib on bleomycin (BLM)-induced pulmonary fibrosis. (A) Evaluation of fibrotic changes in the lung using a numeric fibrotic score. A histological examination of the right lung was performed using H&E staining. Data were analyzed using a one-way ANOVA and displayed as mean ± SD of all fields examined in each group. (B) Collagen contents in left lung lobes

were measured using a hydroxyproline colorimetric assay. Data were analyzed using a one-way ANOVA and displayed as means ± SD in each group. The number of mice used for analysis in each group is shown in the figure as “n”. Data are representative of two separate experiments. *P<0.05, **P<0.01 versus the group treated with BLM alone.

Figure S5.

Anti-fibrotic role of nintedanib in pulmonary fibrosis via the suppression of fibrocyte activity. Fibrocytes and fibroblasts produce fibrotic growth factors such as FGF and PDGF. Growth factor receptors on resident fibroblasts may be activated by fibrocytes or by fibroblasts themselves, and induce the proliferation of fibroblasts. We herein show that nintedanib has three anti-fibrotic roles in pulmonary fibrosis. Nintedanib inhibits the proliferation of fibroblasts induced by fibrocytes, the differentiation of fibrocytes from monocytes, and the migration of fibrocytes. Consequently, nintedanib inhibits the pro-fibrotic activity of fibrocytes.
