# Supplementary material for: Anti-fibrotic efficacy of nintedanib in pulmonary fibrosis via the inhibition of fibrocyte activity
Source: Respir Res. 2017 Sep 15;18:172. doi: 10.1186/s12931-017-0654-2 (PMC5603061; doi:10.1186/s12931-017-0654-2)
Supplement: Supplementary file 2 — Figure S1. Nintedanib inhibits the differentiation of fibrocytes generated from HMNC. Figure S2. Nintedanib did not cause cellular damage in fibrocytes. Figure S3. Histological examination of the anti-fibrotic effects of nintedanib on bleomycin (BLM)-induced lung fibrosis. Figure S4. Quantitative examination of the anti-fibrotic effects of nintedanib on bleomycin (BLM)-induced pulmonary fibrosis. Figure S5. Anti-fibrotic role of nintedanib in pulmonary fibrosis via the suppression of fibrocyte activity. (PDF 884 kb) [file 12931_2017_654_MOESM2_ESM.pdf]

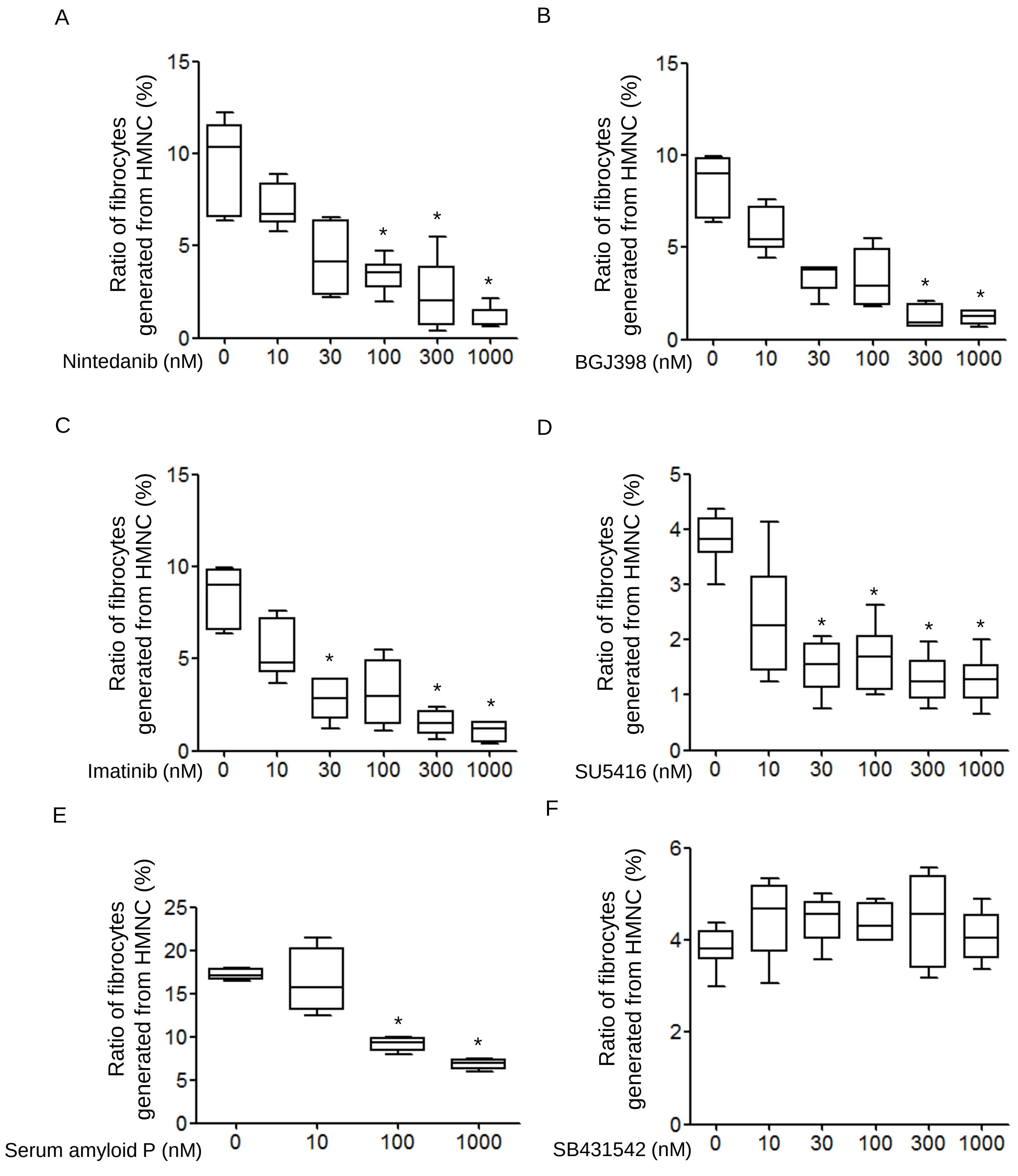
A
Ratio of fibrocytes generated from HMNC (%)
Nintedanib (nM)
0 10 30 100 300 1000
0 5 10 15
B
Ratio of fibrocytes generated from HMNC (%)
BGJ398 (nM)
0 10 30 100 300 1000
0 5 10 15
C
Ratio of fibrocytes generated from HMNC (%)
Imatinib (nM)
0 10 30 100 300 1000
0 5 10 15
D
Ratio of fibrocytes generated from HMNC (%)
SU5416 (nM)
0 10 30 100 300 1000
0 1 2 3 4 5
E
Ratio of fibrocytes generated from HMNC (%)
Serum amyloid P (nM)
0 10 100 1000
0 5 10 15 20 25
F
Ratio of fibrocytes generated from HMNC (%)
SB431542 (nM)
0 10 30 100 300 1000
0 2 4 6

Figure S1

G

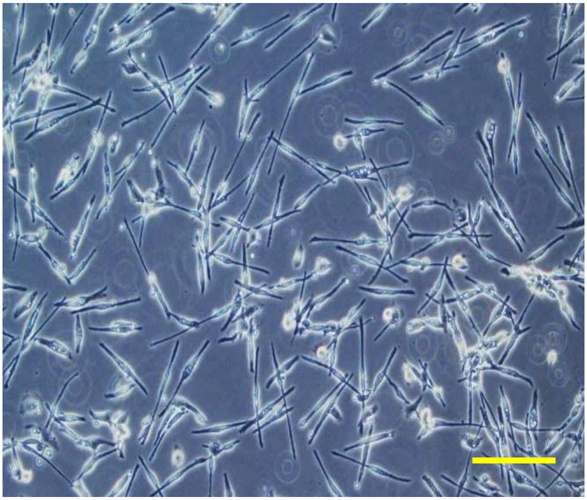

+Vehicle

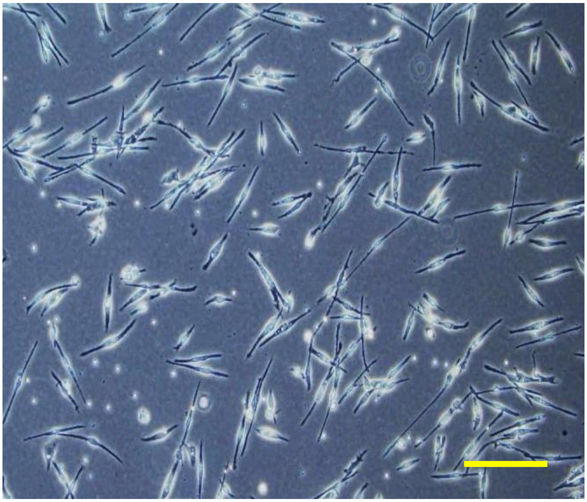

+Nintedanib 10nM

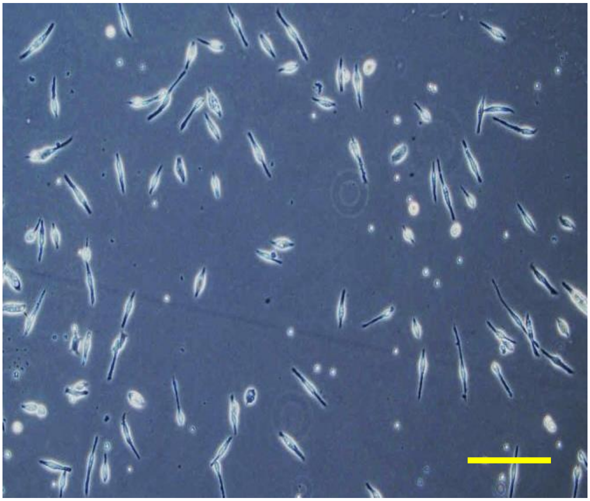

+Nintedanib 100nM

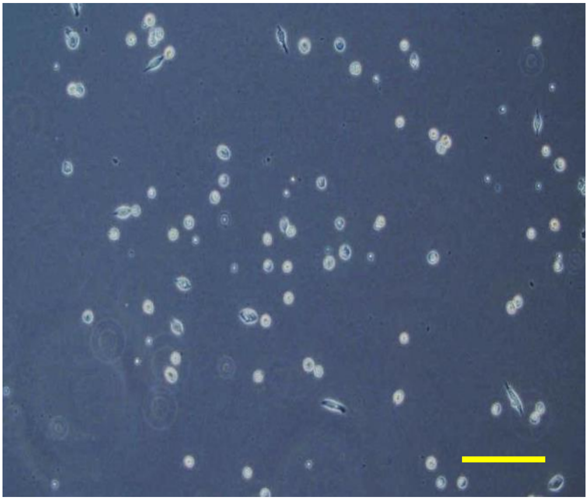

+Nintedanib 1μM

Figure S1

A

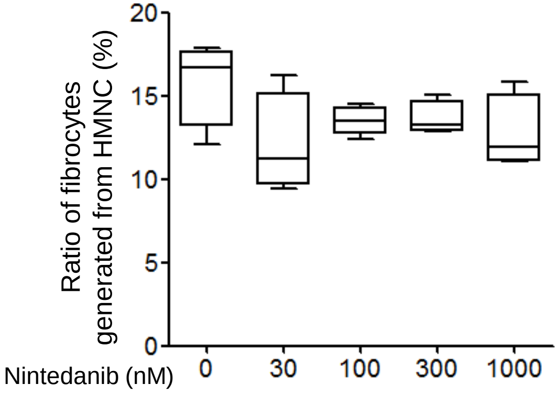
Ratio of fibrocytes generated from HMNC (%)
20
15
10
5
0
Nintedanib (nM)
0
30
100
300
1000

B

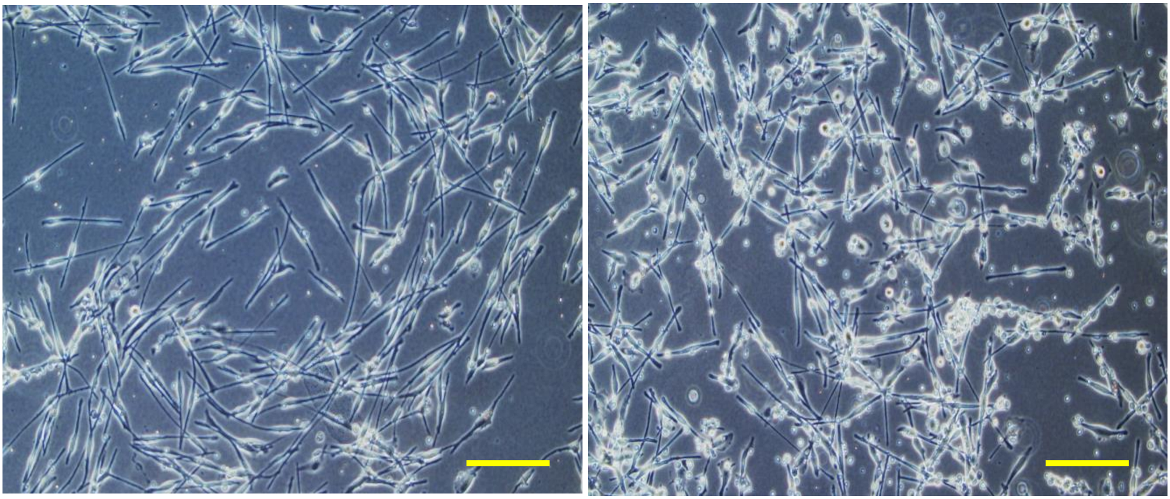
+Vehicle
+Nintedanib 1μM

Figure S2

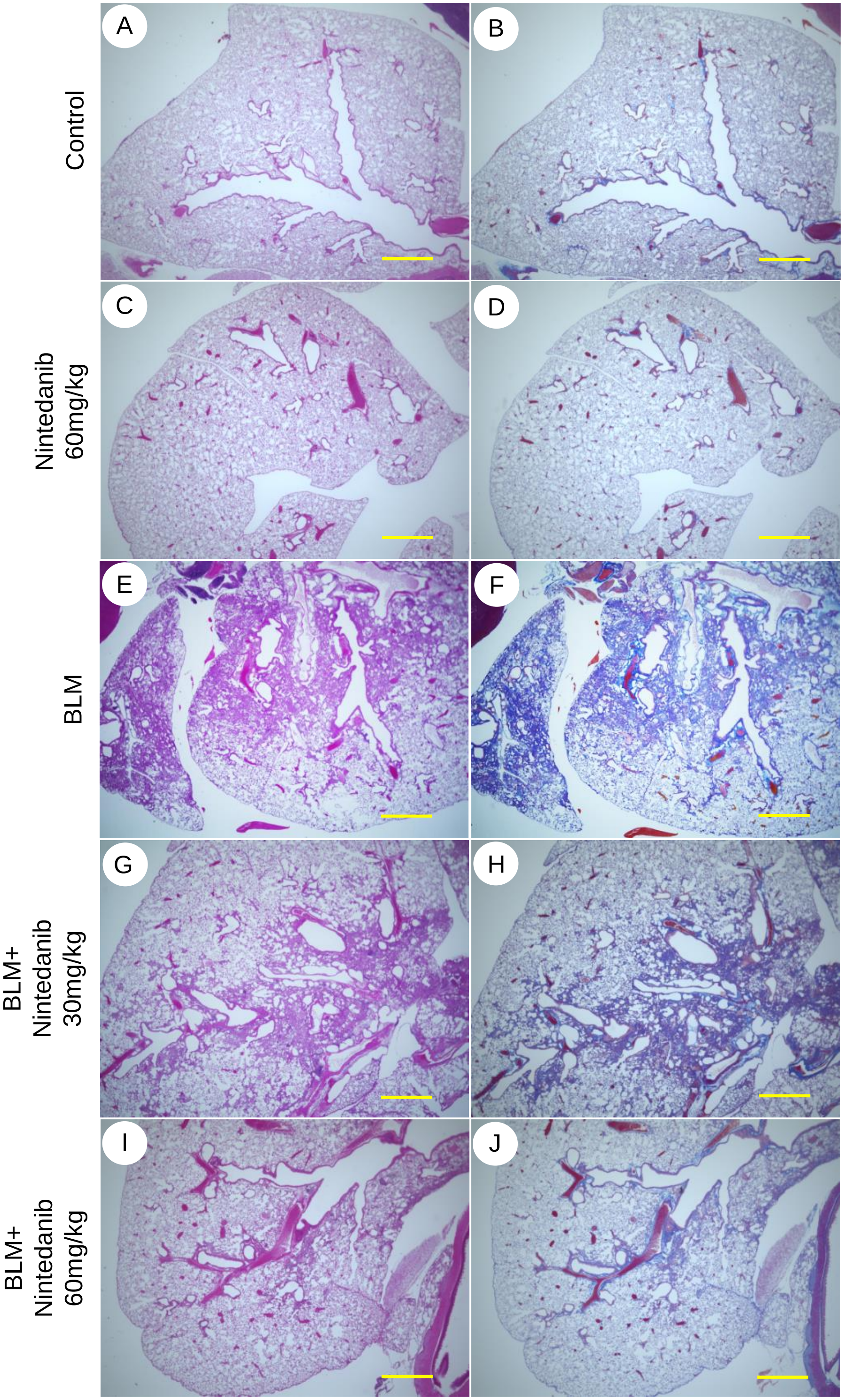
A
B
C
D
E
F
G
H
I
J
Control
Nintedanib 60mg/kg
BLM
BLM+ Nintedanib 30mg/kg
BLM+ Nintedanib 60mg/kg

Figure S3

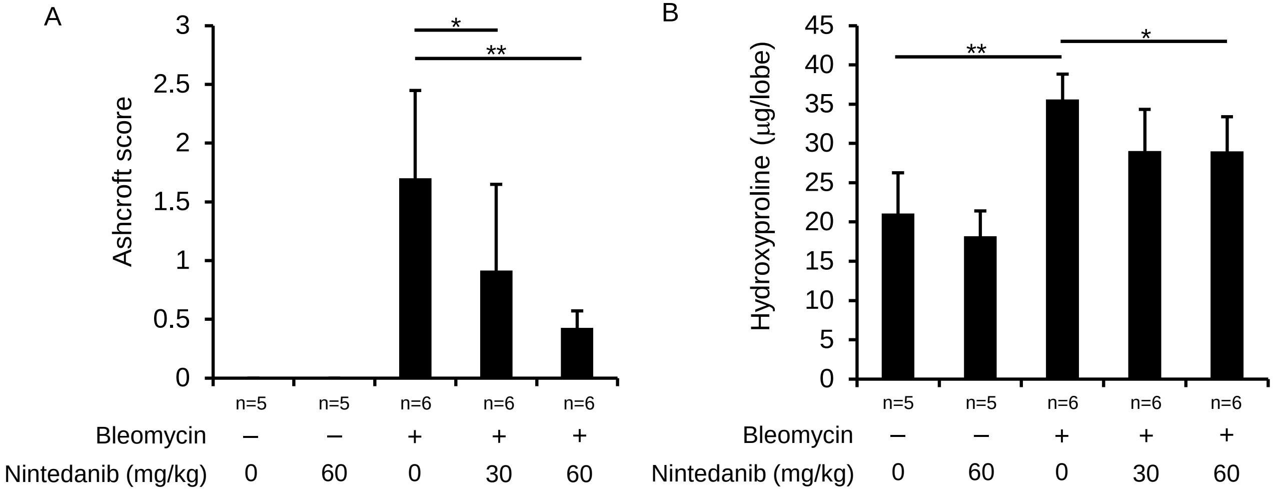
A
B
Ashcroft score
Hydroxyproline (μg/lobe)
*
**
n=5
n=6
Bleomycin
Nintedanib (mg/kg)

Figure S4

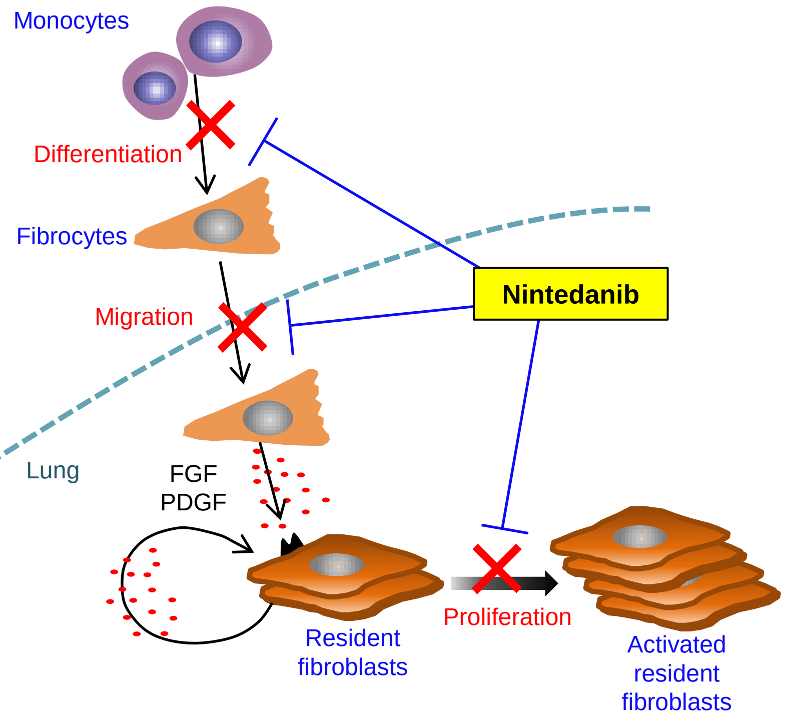

Monocytes
Differentiation
Fibrocytes
Migration
Nintedanib
Lung
FGF
PDGF
Resident fibroblasts
Proliferation
Activated resident fibroblasts

Figure S5
